# Speaker-Versus Listener-Oriented Disfluency: A Re-examination of Arguments and Assumptions from Autism Spectrum Disorder

**DOI:** 10.1007/s10803-017-3215-0

**Published:** 2017-06-20

**Authors:** Paul E. Engelhardt, Oliver Alfridijanta, Mhairi E. G. McMullon, Martin Corley

**Affiliations:** 1 School of Psychology, University of East Anglia, Norwich Research Park, Norwich, NR4 7TJ UK; 2 University of Northumbria, Newcastle upon Tyne, UK; 3 University of Edinburgh, Edinburgh, UK

**Keywords:** Speech fluency, Verbal intelligence, Executive function, Disfluencies, Individual differences

## Abstract

We re-evaluate conclusions about disfluency production in high-functioning forms of autism spectrum disorder (HFA). Previous studies examined individuals with HFA to address a theoretical question regarding speaker- and listener-oriented disfluencies. Individuals with HFA tend to be self-centric and have poor pragmatic language skills, and should be less likely to produce listener-oriented disfluency. However, previous studies did not account for individual differences variables that affect disfluency. We show that both matched and unmatched controls produce fewer repairs than individuals with HFA. For silent pauses, there was no difference between matched controls and HFA, but both groups produced more than unmatched controls. These results identify limitations in prior research and shed light on the relationship between autism spectrum disorders and disfluent speech.

**Electronic supplementary material:**

The online version of this article (doi:10.1007/s10803-017-3215-0) contains supplementary material, which is available to authorized users.

## Introduction

Individuals with high-functioning forms of autism spectrum disorders (HFA) tend to have a self-centric approach to dialogue and poor pragmatic skills.^1^ Thus, they often do not have language impairments per se but do have impairments in pragmatic aspects of language use, as well as atypical prosody (for reviews see, McCann and Peppe 2003; Paul et al. 2005; Tager-Flusberg et al. 2005). In past research (e.g. Lake et al. 2011), it has been argued that if certain types of disfluency are solely (or primarily) for the benefit of the listener or listener-oriented (i.e., in some way helpful to communicative goals), then these disfluencies should be absent in HFA. A classic example of *listener-oriented* disfluency is filled pauses, such as *um* and *uh*, which have been argued to fulfil a variety of discourse-related functions (e.g. holding the floor between turn taking) (Shriberg 1994). In contrast, disfluencies that are *speaker-oriented* are assumed to be due to a variety of speaker-internal factors related to difficulties in language production (e.g. word retrieval difficulty). Returning to the issue of disfluency production in HFA, the key theoretical issue is to determine which types of disfluency are listener-oriented and which are speaker-oriented. In this case, a clinical population has been used to argue a basic theoretical question in psycholinguistics related to speech disfluencies. In the current study, we investigated speech disfluencies in a sample of individuals with HFA and two samples of control participants. The main goal of the current study was to re-examine some of the mixed findings in the existing literature concerning the patterns of disfluency in HFA. In addressing this goal, we note several limitations of prior work that, we argue, has made it difficult to conclude whether people with HFA have different patterns of disfluency compared to their typically-developing peers. Our results also have implications for the clinical literature concerning atypical speech in HFA.

### Types of Disfluency

The main types of disfluency that have been investigated are pauses, repetitions, and repairs (Arnold et al. 2004; Barr 2001; Bortfeld et al. 2001; Deese 1984; Engelhardt et al. 2011; Fox Tree and Clark 1997; Maclay and Osgood 1959; Nooteboom 1980; O’Connell and Kowal 2005; Shriberg 1996). As mentioned previously, most often investigated in the context of “listener-oriented” disfluency are filled pauses, such as *uh* and *um*. Clark and Fox Tree (2002) argued that filled pauses are produced by speakers as a collateral signal of an imminent delay in speech (see also, Brennan and Williams 1995). According to Clark and Fox Tree, *uh* is a signal of an upcoming short delay and *um* is a signal of an upcoming long delay. The second main type of disfluency is repetitions. These occur when the speaker stops speaking and immediately repeats something s/he just said. The literature is not entirely clear whether repetitions are speaker- or listener-oriented. Clark and Wasow (1998) argued for a *continuity hypothesis*, which assumes that speakers repeat material in order to restore continuity to an interrupted constituent, that is, it is easier for the speaker to produce a full constituent rather than a partial phrase or fragment. Repairs also referred to as false starts or revisions occur when the speaker suspends articulation and corrects (or otherwise restarts) with a new word or phrase. Finally, silent or unfilled pauses may be interpreted as disfluencies, although they may also serve rhetorical or other purposes in fluent speech (see Ferreira 2007; Fox Tree 1995, for discussion).

### Disfluency in Attention-Deficit/Hyperactivity Disorder

One impetus for the current study came from a series of papers that investigated disfluency production in Attention-Deficit/Hyperactivity Disorder (Engelhardt et al. 2010, 2009, 2011, 2012; Zentall 1988). In particular, these papers focused on the role of inhibitory control in sentence production because many of the prominent theories of ADHD focus on deficiencies in behavioural-response inhibition (e.g. Barkley 1997; Barkley and Murphy 2006; Martel et al. 2007; Nigg 2001; Nigg et al. 2007; Pennington and Ozonoff 1996; Schachar et al. 1995; Tannock and Schachar 1996). In the Engelhardt et al. studies, participants saw two pictures and a verb and they had to produce a grammatical sentence. The most robust finding with respect to inhibitory control and disfluent speech was the number of repair disfluencies. Individuals diagnosed with the most severe form of ADHD (i.e. those presenting symptoms of both inattention and hyperactivity–impulsivity—the combined subtype) produced more repairs compared to typically-developing controls (Engelhardt et al. 2010, 2012). Approximately two-thirds of the repairs were cases in which participants made a structural revision, that is, they switched from active to passive voice (e.g. *the girl … the bicycle was ridden by the girl*), and approximately one-third showed clear evidence of a production error (e.g. *the boy … girl had ridden the bicycle*). The latter type is consistent with lexical selection difficulty (Berg and Schade 1992; Shao et al. 2013). These findings were later extended to individual differences in a typically-developing sample. Engelhardt et al. (2013) showed that performance on the Stroop task (Golden 1978; Stroop 1935) and stop-signal reaction time (Logan 1994), both primarily inhibition tasks, accounted for nearly one-third of the variance in repair disfluency production and this finding held even when individual differences in intelligence and set shifting were controlled for. Set shifting refers to the ability to shift back and forth between multiple tasks, operations, or mental sets (Monsell 1996).

These results are relevant to the current study in two ways. The first is that a clinical population was used to examine a basic theoretical question concerning the role of executive functioning in the fluency of speech outputs. The second is that these studies identified a robust relationship between inhibitory control and repairs. One issue that we note in the ASD-disfluency literature is that many of the existing studies did not take into account differences in (verbal) intelligence and executive function (Hill 2004), and thus, these studies overlooked a critical factor that has been previously shown to influence the fluency of language outputs.

### Disfluency in Autism Spectrum Disorder

As mentioned above, there has been growing interest in the types and rates of disfluency production in individuals with HFA (Scott 2015). Several studies have reported differences between HFA and typically-developing controls (Shriberg et al. 2001; Suh et al. 2014; Tager-Flusberg et al. 2005; Thurber and Tager-Flusberg 1993). Table 1 contains a summary of the published studies broken down by disfluency and task type.^2^ The summary in Table 1 shows that results have been mixed. In the remainder of this section, we review these results with a particular focus on the conflicting data and identifying limitations in prior work. A key study, which motivated the current one, was conducted by Lake et al. (2011). Those researchers investigated *speaker-oriented* versus *listener-oriented* disfluency, and the rationale behind the study focused on the fact that individuals with HFA tend to operate more self-centrically in dialogue and have difficulty with social interactions. Thus, Lake et al. hypothesized that individuals with HFA should produce fewer *listener-oriented* (or helpful) disfluencies, and in cases where individuals with HFA produce fewer disfluencies than typically-developing controls, those types of disfluency were assumed to be listener oriented. Conversely, in cases where individuals with HFA produce more disfluency, those disfluencies were assumed to be speaker oriented (i.e. related to speaker-internal factors).

**Table 1:** Summary of disfluency production comparing individuals with ASD to typically-developing controls

Study	Sample size	Filled	Unfilled	Repetitions	Repairs	Task
Irvine et al. (2016)	(ASD = 24, TD = 16)	ASD < TD^a^	*NA*	*NA*	*NA*	Monologue (painting descriptions)
Lake et al. (2011)	(ASD = 13, TD = 13)	ASD < TD	ASD > TD	ASD > TD	ASD < TD	Dialogue (question answering)
Shriberg et al. (2001)	(ASD = 30, TD = 53)	*NA*	ASD > TD	ASD > TD	ASD > TD	Dialogue (ADOS interview)
Suh et al. (2014)	(ASD = 15, TD = 15)	*NS*	*NA*	ASD > TD	ASD > TD	Monologue (story telling)
Thurber and Tager-Flusberg (1993)	(ASD = 10, TD = 10)	*NA*	ASD < TD^b^	*NS*	*NS*	Monologue (story re-telling)

*NA* not analyzed/available, *NS* not significant

^a^Significant differences observed in production of um’s but not uh’s

^b^Significant differences in non-grammatical pauses but not grammatical pauses

In the Lake et al. (2011) study, data consisted of 5–10 min conversations in which a trained experimenter asked participants questions about their hobbies and interests. Lake et al. found that individuals with HFA produced fewer filled pauses and repairs, and more unfilled pauses and repetitions compared to controls (see Table 1). On the basis of those results, Lake et al. concluded both filled pauses and repairs are types of listener-oriented disfluency and that the speech of individuals with HFA is less “listener-oriented”. Also, because individuals with HFA produced more repetitions, Lake et al. argued that repetitions are not a listener-oriented attempt to restore fluency, but instead, are an automatic outcome of detecting and correcting problems in one’s own speech. However, there were several weaknesses in this study. First, individuals with HFA had a tendency to produce one word answers and often needed prompting (i.e., re-asking or re-phrasing of questions in order to elicit sufficient responses). Second, the groups were matched on age and gender, but not on intelligence or education. The absence of intelligence measures, and in particular verbal intelligence, is problematic given the strength of the relationship between verbal intelligence and repetitions that has been noted in previous work. Third, there were differences in mean length of utterance. Individuals with HFA produced fewer words overall compared to controls. We return to this issue below when we discuss differences between controlled and naturalistic production tasks. A similar study, which also utilized interactive dialogue, was conducted by Shriberg et al. (2001). Their results for unfilled pauses and repetitions were consistent with Lake et al., but repairs showed the opposite pattern (ASD > TD). However, the Shriberg et al. study suffers from many of the same problems, in that participant groups were not well matched. In Shriberg et al., participants were only matched on age.

In a more recent study, Irvine et al. (2016) used a monologue task in which participants were required to describe 12 different paintings. Each description was approximately 10 s long and a number of the trials required concurrent finger tapping. In this study, the authors focused exclusively on filled pauses to examine a similar research question as Lake et al. (i.e., Do individuals with HFA produce *listener-oriented* disfluency?). Their results showed only a difference in the rates of *um* production, and importantly, this difference was linked with ASD symptom severity. The Irvine et al. study was methodologically more robust because it also assessed several executive functions, as well as language ability. Their groups did not differ in age, gender, and non-verbal intelligence, but were marginally different in verbal intelligence.^3^

The final two studies (Suh et al. 2014; Thurber and Tager-Flusberg 1993) used a monologue story-telling task. In Suh et al. (2014), an examiner gave the participant a picture book and started a story, and the participant was asked to finish it. The stories ranged in length from 127 to 576 words, but importantly, there were no significant differences between groups in terms of number of words, number of utterances, or mean length of utterance (MLU). In addition, the groups were not significantly different in age, gender, or verbal intelligence, but non-verbal intelligence was marginally significant (ASD < TD). Suh et al. reported significant group differences for repetitions and repairs, and for both, the group with an ASD produced more disfluencies than the typically-developing group. These finding are consistent with Shriberg et al. (2001). The final study by Thurber and Tager-Flusberg (1993) looked at story re-telling, and in this study, there were differences between groups in the length of the narratives produced (differences in MLU and fewer propositions). The fact that the stories differed in length and quality is problematic from an empirical point of view because the cognitive demand of the speaking task is different. Because typically-developing participants produced more complex and intricate stories, the task demands for them were higher, and as such, disfluency rates are expected to be greater irrespective of the needs of listener.

### Controlled Versus Naturalistic Production

Tasks used to study language production can be classified into two broad categories: controlled and naturalistic. Controlled production tasks are designed to elicit specific responses, and these tasks tend to be monologue as opposed to dialogue. For example, in sentence production, participants may be primed to produce alternating forms of a sentence, such as *Joe handed the microphone to Bill* versus *Joe handed Bill the microphone* (Pickering and Branigan 1998). These sentence production tasks typically require participants to either repeat a complex sentence or to produce a grammatical utterance by describing a picture or event (Engelhardt et al. 2009; Myachykov et al. 2012; Oram et al. 1999; Redmond 2004). In contrast, naturalistic tasks typically have participants engage in an activity or conversation, which is recorded, and then the recordings are analyzed for factors, such as number of interruptions, total number of words/utterances produced, grammaticality mistakes, disfluencies, etc. (e.g. Scott and Windsor 2000; Zentall et al. 1983). The advantage of naturalistic tasks is that they more closely mirror everyday language use, especially tasks that involve interactive dialogue. However, a major disadvantage is that they suffer from a lack of control over both the content of speech and other situational factors that could potentially affect what and how things are said, which leads to a range of potential confounds (see Lake et al. 2011; Shriberg et al. 2001).

In the current study, our aim was to maintain control over the task demands associated with speaking where possible. For this reason, participants produced the same words and the same syntactic structures, which ensured that task demands were equal for both the group with HFA and typically-developing controls.

### Current Study

In much of the past research, the relationship between disfluency production and individual differences variables was negative, that is, lower-ability individuals produce more disfluencies (e.g. Engelhardt et al. 2010). These negative relationships were found both in clinical populations (e.g. Shriberg et al. 2001) and in typically-developing individuals (Engelhardt et al. 2013). (The results from the HFA studies are summarized in Table 1, and the ADHD results are summarized in the supplementary material.) In the current study, we investigated differences in disfluency production between HFA and two groups of typically-developing controls. One group of controls was matched in terms of age and gender, and the second was randomly selected from a larger study that used the same protocols. Like Irvine et al. (2016), we sought to control for a range of individual differences variables. In cases where we observed significant group differences, we also looked at whether the differences could be explained by any of the individual differences variables in our dataset. Thus, the goals of this study were to provide some clarification on (1) the theoretical question regarding speaker- versus listener-oriented disfluency, (2) the broader literature of atypical speech in HFA, and (3) the role of individual difference variables in disfluency production. As reviewed above, many of the previous HFA studies showed mixed findings. These differences may be in part due to differences in the tasks used, and the fact that control groups were not matched on key variables. We chose a controlled sentence production task in which participants had to memorize and then repeat back a complex sentence. The sentences were recorded and coded for the different types of disfluency (i.e. filled and unfilled pauses, repetitions, and repairs). We expected that individuals with HFA would produce fewer filled pauses and more repetitions. These types of disfluency have been relatively consistent in previous literature (see Table 1). Effects of unfilled pauses and repairs were less consistent in previous research, but given the ADHD work (e.g. Engelhardt et al. 2010), we expected both to be produced more frequently in individuals with HFA.

## Method

### Participants

Participants were 39 adults, 13 with HFA and 26 typically-developing controls. Thirteen control participants were recruited and tested to serve as age and gender matched controls for the group with HFA. We randomly selected 13 further typically-developing controls from a larger study to serve as an unmatched control group. The participants with HFA were compensated £20 for taking part in the study, and approximately one-quarter of the typically-developing participants received £20 and three-quarters received credits for the undergraduate psychology pool at Northumbria University. Table 2 contains a summary of demographic data and descriptive statistics for the standardized measures. Three participants with HFA, also reported a concurrent diagnosis of ADHD, and two reported a learning disability.

**Table 2:** Demographic data, verbal intelligence, working memory, and autism quotient broken down by the three groups

	ASD (13)	Matched (13)	Unmatched (13)	One-way ANOVA	Significant *t-*tests (*p* < .05)^a^		
Demographic	Mean (SD)	Mean (SD)	Mean (SD)		ASD-Mat.	Mat.-Unmat.	ASD-Unmat.
Age (years)	26.33 (10.97)^b^	21.5 (4.54)	19.7 (.84)	*F* = 3.22, *p* = .052			
Gender (% male)	69%	31%	8%	*F* = 7.00, *p* < .01			ASD > Unmat.
Education attained^c^	3.09 (2.26)	3.46 (.97)	3.12 (.87)	*F* = .26, *p* = .77			
WAIS verbal comprehension							
Vocabulary	35.77 (16.18)	37.38 (9.10)	39.15 (5.83)	*F* = .30, *p* > .70			
Similarities	19.08 (4.46)	21.85 (4.02)	23.31 (3.68)	*F* = 3.63, *p* < .05			ASD < Unmat.
Information	16.00 (5.87)	14.85 (3.63)	15.46 (3.82)	*F* = .21, *p* > .80			
Comprehension	13.23 (4.94)	19.08 (4.97)	15.08 (5.07)	*F* = 4.65, *p* < .05	ASD < Mat.		
Mean	21.02 (7.85)	23.29 (4.62)	23.25 (3.49)	*F* = .72, *p* > .40			
WAIS working memory							
Arithmetic	13.31 (2.90)	14.08 (3.14)	13.69 (2.56)	*F* = .23, *p* > .70			
Digit span	9.38 (1.89)	10.54 (1.94)	11.31 (1.80)	*F* = 3.45, *p* < .05			ASD < Unmat.
Backward digit span	6.23 (2.39)	6.62 (1.71)	8.08 (1.93)	*F* = 2.99, *p* = .063			ASD < Unmat.
Mean	9.64 (1.92)	10.41 (1.81)	11.03 (1.49)	*F* = 2.04, *p* > .10			ASD < Unmat.
Autism quotient							
Social skill	5.00 (2.68)	2.15 (2.15)	.85 (.99)	*F* = 13.76, *p* < .01	ASD > Mat.		ASD > Unmat.
Attention switching	6.62 (1.85)	5.46 (1.56)	3.77 (2.01)	*F* = 8.09, *p* < .01		Mat. > Unmat.	ASD > Unmat.
Attention to detail	6.54 (2.11)	5.62 (1.33)	3.85 (2.04)	*F* = 7.07, *p* < .01		Mat. > Unmat.	ASD > Unmat.
Communication	5.46 (2.40)	2.92 (1.38)	1.92 (1.66)	*F* = 12.45, *p* < .01	ASD > Mat.		ASD > Unmat.
Imagination	4.31 (2.06)	2.31 (1.70)	2.23 (1.64)	*F* = 5.51, *p* < .01	ASD > Mat.		ASD > Unmat.
AQ (total)	27.92 (8.32)	18.62 (5.38)	12.62 (3.66)	*F* = 20.80, *p* < .01	ASD > Mat.	Mat. > Unmat.	ASD > Unmat.

^a^Paired-comparisons were independent samples t-tests

^b^One participant did not report their age

^c^Number of years of education beyond age 16

### Standardized Measures

#### Intelligence and Working Memory

Participants completed seven subtests from the Wechsler Adult Intelligence Scale 3rd edition (Wechsler 1997). The verbal intelligence subtests were comprehension, information, similarities, and vocabulary, and the working memory subtests were arithmetic, backward digit span, and digit span.

#### Autism Spectrum Quotient

The autism quotient assesses autism spectrum traits, and consists of 66 items (Baron-Cohen et al. 2001; Bishop et al. 2004). It contains five subscales: social skill, attention switching, attention to detail, communication, and imagination.

### Sentence Production

#### Materials

The 40 experimental items were taken from Christianson et al. (2001) and Ferreira et al. (2001). Each sentence contained a main clause and a subordinate clause, and the order was reversed on half of the items. None contained commas separating the subordinate and main clauses. There were 421 words in total in the critical items.

#### Procedure

The task was based on the procedure from Ferreira (1991) (see also, Bock 1996; Bock and Levelt 1994; Ferreira and Engelhardt 2006; Levelt 1989). Participants were instructed that they would see a sentence that they had to memorize and repeat back, and that it was important that they spoke the sentence exactly as it was written and in a natural manner. Participants pressed the space bar and a fixation cross appeared for 1 s. The fixation cross was followed by the sentence, and it was presented in the centre of the computer screen. After participants had memorized the sentence, they pressed the space bar, and a question appeared on the screen (i.e. “What happened?”). Participants spoke the sentence out loud, and when they were finished speaking they pressed the space bar to start the next trial. There were three practice trials and 40 experimental items. The order of trials was randomly determined for each participant. If participants forgot the sentence on a particular trial, they could press the “R” key to go back and re-view the sentence. Partial recordings were not saved. Participants spoke into a condenser microphone in a sound dampened testing cubicle and the experiment was programmed with E-prime experimental software. The sentences were automatically recorded and saved as .wav files.

### Utterance Coding

#### Recall Errors

Any errors in the utterance affecting content words were counted as recall errors. These included omissions of content words, incorrect inclusions, and incorrect substitutions (e.g. *archivist* vs. *activist, large* vs. *big, floor* vs. *ground*, etc.). Minor differences (e.g. *eating* vs. *eatin, book* vs. *books*) and differences involving function words (e.g. *the* vs. *a, have* vs. *has*) were not counted as recall errors.

#### Disfluency

**Fig. 1:**
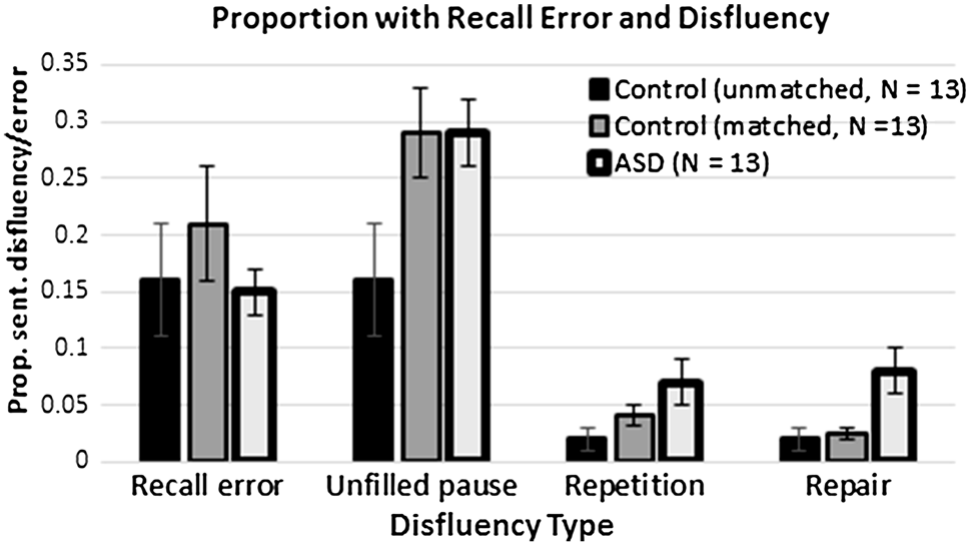
Proportion of recall errors and disfluency per sentence broken down by diagnostic group. *Error bars* show the standard error of the mean

Four main types of disfluency were examined: filled pauses, unfilled pauses, repetitions, and repairs.^4^ Repetitions refer to unintended repeats of a word or string of words with no functional benefit. Repairs occur when a speaker suspends articulation, and then starts over with a new word or phrase. We also assessed the lengths of all unfilled pauses that were 250 ms or greater. We viewed the threshold for an unfilled pause as a somewhat subjective decision because often researchers will utilize a higher threshold (e.g. 1–3 s), so as to exclude prosodic pausing (Kormos and Denes 2004; Lake et al. 2011). However, a recent study by De Jong and Bosker (2013) that investigated perceptions of fluency in L2 learners and accounted for speech rate, argued that 250 ms is the best threshold for unfilled pauses, and this is consistent with the original work of Goldman-Eisler (1968) (see also, Garrett 1982; Harley 2013; Harley and MacAndrew 2001; Redford 2013). With a 250 ms threshold, approximately 30% of sentences contained at least one unfilled pause. The dataset was coded twice, once by the second author and once by a trained research assistant.^5^ The first author compared the two data files and resolved discrepancies. In cases in which the length of an unfilled pause differed by more than 50 ms, it was reassessed by the first author. For the remainder, we averaged the two durations. The corpus contained 1560 sentences (approximately 16,500 words in total), and the dependent variable for the disfluency analysis was proportion of sentences with a particular type of disfluency (see Fig. 1).

### Procedure

Typically-developing participants were recruited via fliers posted on university grounds and by advertisement on the Northumbria University undergraduate participation pool. Participants with HFA were recruited primarily through the University of Newcastle Adult Autism Spectrum Cohort and a local ASD charity. Thus, all participants with HFA had an existing ASD diagnosis. Upon entering the lab, participants provided written informed consent, basic demographic information, and completed the autism spectrum quotient. They then completed each of the tests in the battery (verbal intelligence, working memory, and the sentence production task). The majority of the controls and a few of the participants with HFA also completed computerized versions of both the Wisconsin Card Sort task and the Stroop task, but because these were incomplete datasets, they were not included in the analyses. Tasks were completed in different rooms and in different testing cubicles, and participants were given obligatory breaks between tasks to avoid fatigue. Each participant completed the tasks in the same order. The entire testing session lasted approximately 2 h.

### Data Screening and Preparation

Data points >3.0 standard deviations from the mean for each variable in the data set were defined as outliers. Outliers were replaced with the mean of that variable (McCartney et al. 2006; Stevens 2002; Wilcox 2002; Wilcox et al. 1998). This avoids listwise deletion and the corresponding reduction in power (Shafer and Graham 2002). There were three outliers in the dataset, which were assessed via standardized values. Prior to inferential analyses, the memory and disfluency proportions were transformed using a square root transformation to correct skew (Kline 1998).

## Results

Thirty-eight sentences were not recorded for the participants with HFA and two sentences were not recorded for typically-developing controls due to errors with the experiment programme. Figure 1 shows the mean recall errors and disfluencies as a proportion per sentence produced, and the correlations between variables are presented in Tables 3 and 4.

**Table 3:** Bivariate correlations between verbal intelligence, working memory, and disfluencies

Variable	1	2	3	4	5	6	7	8	9	10	11	12	13	14
1. Age	–	−.05	.38*	.12	.16	−.07	.06	.13	−.13	−.12	−.04	.23	.04	.47**
2. Gender		–	.49**	.14	.21	−.05	.07	.06	.01	−.02	−.05	.14	.02	.14
3. ASD status			–	−.34**	.09	−.39*	−.11	−.10	−.25	−.37*	−.10	.21	*.29* ^#^	.54**
4. Comprehension				–	.64**	.59**	.64**	.28	−.07	.06	.06	.01	−.31*	−.18
5. Information					–	.49**	.77**	.26	−.03	.05	−.03	−.02	−.32*	−.17
6. Similarities						–	.61**	.19	.11	.22	−.15	−.18	−.37*	−.38*
7. Vocabulary							–	.23	−.07	.06	−.05	−.11	−.37*	−.22
8. Arithmetic								–	.22	.45**	−.31*	−.14	−.04	−.14
9. Backward digit									–	.58**	−.41**	*−.28* ^#^	−.21	−.33*
10. Digit span										–	*−.29* ^#^	−.38*	−.25	−.39*
11. Memory errors											–	.45**	.12	.12
12. Unfilled pauses												–	.21	.20
13. Repetitions													–	.67**
14. Repairs														–

Italicized numbers indicate critical correlations for the dependent measures

^#^ *p* < .08, *p < .05, **p < .01. Gender coded 0 = male and 1 = female

**Table 4:** Bivariate correlations between autism spectrum quotient scores and disfluencies

Variable	1	2	3	4	5	6	7	8	9	10	11	12	13
1. Age	–	−.05	.38*	.53**	.47**	.38*	.16	.59**	.45**	−.04	.23	.04	.47**
2. Gender		–	.49**	.27	.12	.26	.19	.32*	.21	−.05	.14	.02	.14
3. ASD status			–	.67**	.63**	.45**	.41*	.62**	.48*	−.10	.21	*.29* ^#^	.54**
4. AQ total				–	.89**	.84**	.60**	.78**	.70**	−.02	*.28* ^#^	.21	.52**
5. Social skill					–	.67**	.46**	.68**	.53**	.02	.25	.25	.48**
6. Attention switching						–	.40*	.56**	.61**	.03	*.31* ^#^	.22	.40*
7. Attention to detail							–	.26	.21	−.20	.06	.08	.20
8. Communication								–	.41**	−.05	.19	.17	.56**
9. Imagination									–	.14	.23	.09	.34*
10. Memory errors										–	.45**	.12	.12
11. Unfilled pauses											–	.21	.20
12. Repetitions												–	.67**
13. Repairs													–

Italicized numbers indicate critical correlations for the dependent measures

^#^ *p* < .08, *p < .05, **p < .01. Gender coded 0 = male and 1 = female

### Recall Errors

A between subjects one-way ANOVA showed that there were no differences between groups in the number of recall errors *F*(2,36) = .38, *p* = .68. Thus, there were no differences in terms of recall accuracy between the three groups.

### Disfluency

Across the entire dataset there were only three filled pauses produced, and thus, there was not a sufficient number for an inferential analysis. For unfilled pauses, a one-way ANOVA showed there was a significant difference between groups *F*(2,36) = 5.27, *p* = .01. Paired comparisons revealed that the matched and unmatched controls were significantly different *t*(24) = 2.46, *p* = .02, as were the group with HFA and the unmatched controls *t*(24) = 2.81, *p* = .01. The matched control group and the group with HFA were not significantly different (*p* > .80). For repetitions, there were no significant differences *F*(2,36) = 1.95, *p* = .16.^6^ Finally, for repairs there was a significant difference between groups *F*(2,36) = 6.37, *p* = .004. The group with HFA was significantly different from both the matched *t*(24) = −2.63, *p* = .02 and unmatched groups *t*(24) = 3.02, *p* = .01. The two control groups were not significantly different from one another (*p* > .40). As can been seen in Fig. 1, the group with HFA produced more repairs compared to controls.

### Individual Differences Variables

A further goal of the current study was to investigate how individual difference variables relate to rates of disfluency production. We noted the lack of between group control variables as a potential weakness in some previous work. We believe that our test battery allows some further insights, and thus, helps resolve (at least some) of the conflicting findings outlined in the Introduction and summarized in Table 1. The bivariate correlations, which are presented in Tables 3 and 4, reveal some interesting patterns. Based on a series of studies published by Engelhardt and colleagues (summarized in the supplementary materials), we expected correlations between individual differences variables and disfluencies to be negative, and in the range of .20 to .30.

In the current study, the matched controls and the group with HFA produced unfilled pauses at almost exactly the same rate (approximately one in three sentences had an unfilled pause). However, the unmatched controls produced significantly fewer unfilled pauses. Lake et al. and Shriberg et al. reported that individuals with HFA produce more unfilled pauses. In the current study, unfilled pauses correlated significantly with one of the memory subscales (digit span) and marginally correlated with backward digit span. Both were negative. There was also a significant (positive) correlation between recall errors and unfilled pauses. Given the fact that the memorize-and-repeat task used in this study primarily taxes memory resources, we think that the unfilled pauses in this task reflect retrieval problems (i.e. people pause because they are in the process of retrieving information from memory).^7^ Interestingly, the effect of group on unfilled pauses remained significant even with digit span covaried *F*(2,35) = 3.33, *p* = .05, which suggests that ASD status contributes unique variance, that is, the group differences are not simply accounted for by the memory ability differences between groups.

In terms of repetitions, we did not observe significant differences. In fact, the proportions were nearly identical to those reported by Thurber and Tager-Flusberg. We found that repetitions correlated most highly with verbal intelligence (*r’s* between −.31 and −.37) (see Table 3). Three of the previous ASD studies reported that individuals with HFA produce more repetitions than controls. However, when considering the strength of the correlations between repetitions and verbal intelligence, and the fact that our groups had comparable verbal intelligences, we contend that the reported effect of repetitions in Lake et al. (2011) and Shriberg et al. (2001) is due to a confound, specifically that the groups in those studies were not assessed/matched on verbal intelligence. The one exception is the study by Suh et al. (2014). Those authors did match their groups on verbal intelligence, and did report significant differences the number of repetitions. One possible explanation concerns the power of the study, as Suh et al. did have a substantially larger sample. Another possibility is differences between the tasks used in the two studies. We return to both of these issues in the “Discussion”.

As can be seen in Table 3, repairs correlated significantly with age and ASD status, and three of the seven WAIS subscales (i.e. similarities, backward digit, and digit span). As a follow up, we re-ran the between group analysis on the number of repairs in which we covaried age, similarities, and the span subscales. The significant effect of diagnostic group on repairs held even when age *F*(2,34) = 3.71, *p* = .04, similarities *F*(2,35) = 3.41, *p* = .04, digit span *F*(2,35) = 4.05, *p* = .03, and backward span *F*(2,35) = 5.19, *p* = .01, were covaried. The correlations with the AQ also revealed several significant correlations with the number of repairs. Thus, across both sets of bivariate correlations, there is a robust relationship between HFA (and ASD symptoms) and the increased tendency to produce repair disfluencies.

## Discussion

Previous studies have used individuals with HFA in order to test a hypothesis concerning listener- versus speaker-oriented disfluency. The rationale is that individuals with HFA tend to have poor social interactions and operate self-centrically in conversation, and as a result, they should fail to show types of disfluency that are listener-oriented. In contrast, if individuals with HFA produce more disfluencies of a particular type, then these disfluencies are assumed to be speaker-oriented (i.e. due to speaker-internal factors). According to Lake et al. (2011), filled pauses and repairs showed a listener-oriented pattern (ASD < TD) and unfilled pauses and repetitions showed a speaker-oriented pattern (ASD > TD). However, there has been a lot of mixed findings in the literature. In the current study, we found significant differences in the number of repairs and unfilled pauses, and the pattern was consistent with the findings of Shriberg et al. (i.e. ASD > TD). We also found several significant (positive) correlations between repairs and AQ scores, which further confirms an association between ASD and the tendency to produce more repair disfluencies. The group effect on repairs was robust even after covarying all significant individual differences variables (i.e. age, similarities, digit span, and backward digit span). To our knowledge, the results concerning the relationship between repairs and working memory is a novel finding, but at this point, we do not know whether this relationship is unique to our task which relied heavily on memory for successful performance. In any event, the difference between individuals with HFA and controls was robust with memory differences controlled, and as, such is not simply explained by individual differences in working memory ability.

We did not observe a significant difference in repetitions. However, like in the ADHD studies (e.g. Engelhardt et al. 2010), we observed significant correlations between repetitions and verbal intelligence. In the “Results” section, we argued that the Lake et al. and Shriberg et al. findings with respect to repetitions is very likely due to fact that those studies did not assess/control for individual differences in verbal intelligence. We do note however, that the trend in our data and the trend in the Thurber and Tager-Flusberg study are in the same direction as results reported in the three studies that did report significant differences between groups. Thus, for repetitions there is a consistent pattern in which individuals with HFA produce numerically more repetitions. The one study that does not fit our verbal intelligence explanation is Suh et al., they reported that individuals with HFA produced significantly more repetitions. The groups in that study were not significantly different in verbal intelligence but the means were HFA = 102 versus TD = 112. The lack of significant differences is no doubt partially due to the smallish sample sizes in the existing studies, and the large range in verbal intelligence. Unfortunately, Suh et al. did not report the correlations (or partial correlations) concerning the relationship between ASD status, verbal intelligence, and repetitions. We suspect that the group effect on repetitions in Suh et al. would not remain if verbal intelligence was covaried. Thus, it is our conclusion that autism spectrum disorders are not associated with an increased tendency to repeat material when differences in verbal intelligence are taken into account.

We also observed differences in terms of unfilled pauses, but the pattern was such that the matched controls and the group with HFA were not significantly different but both were different from the unmatched controls. Two previous studies reported that individuals with HFA produced more unfilled pauses than controls. Again, those two studies are the ones that did not match their groups particularly well (i.e. Lake et al. and Shriberg et al.). Thurber and Tager-Flusberg reported different patterns for what they classified as grammatical versus non-grammatical pauses. ASD participants produced more grammatical pauses but fewer non-grammatical pauses, which does not make sense, especially given the conclusions of Lake et al. and findings from the ADHD literature. One issue with unfilled pauses is that the criteria (or threshold) used for determining unfilled pauses varies between studies. Lake et al. used a particularly long threshold (3 s). In the current study, we found that unfilled pauses were correlated with the digit span subscale. We note that the rate of unfilled pauses in the unmatched sample was approximately one pause in every six sentences, substantially lower than one-in-three observed in the other two groups. Moreover, the unmatched controls had significantly higher memory abilities compared to the group with HFA (see Table 2), but the effect of group on unfilled pauses remained even with digit span covaried. However, despite this, we are still sceptical of findings from unmatched samples (i.e. our differences turned on the matched group).

Several issues are worth raising before we dig into the differences between tasks and the theoretical implications of this research. The first is that we did not observe many filled pauses, and thus, there were not enough for a statistical analysis. This is unfortunate because filled pauses are often claimed to be a listener-oriented type of disfluency (e.g., Clark 1994; Clark and Fox Tree 2002). Thus, the expectation for filled pauses is reversed (i.e. higher functioning individuals should produce more). The second concerns unfilled pauses. As just mentioned, the criteria for unfilled pauses varies between studies, and so, any comparisons between studies requires substantial caution. The third concerns the memorize-and-repeat task we used. We classified errors in the verbal productions that our participants produced as “recall errors”. However, as one reviewer correctly pointed out, the task does not actually distinguish between errors at encoding (i.e. in reading the sentence) and errors in memory recall. This is especially true of recall errors such as *archivist* versus *activist*. Related to this issue, we did not include a language ability assessment in our test battery, and some studies report that individuals with HFA do have difficulty with some aspects of morphology and syntax (Brynskov et al. 2017; Park et al. 2012). We acknowledge the lack of language ability as a limitation of our study, but at the same time, there are several aspects of our data which we think makes this less of a concern. First, the group with HFA produced fewer recall errors than controls. Second, there were no significant differences in terms of the level of education (see Table 2). Third, there were relatively few differences between groups in terms of verbal intelligence, and verbal intelligence has recently been shown to be a strong predictor of syntactic ambiguity resolution, which is one of the most difficult syntactic processing operations to overcome (Engelhardt et al. 2017; Van Dyke et al. 2014).

### Controlled Versus Naturalistic Production

Prior studies have used a variety of different speaking tasks: They range from fully interactive dialogue to essentially scripted monologue. In the Introduction, we outlined the pluses and minuses of each type of task. On the one hand, the variability in tasks may seem problematic or a limitation when it comes to between study comparisons. On the other hand, if the results for different types of disfluency are consistent across tasks, then it would support generalizability. We see the variability in the literature as a strength rather than a limitation, and in cases, where results are not consistent, we look to differences in tasks and in task demands to account for conflicting findings. We chose a controlled production task as we were particularly keen to ensure that the task demands were equal between the different groups.

We found that unfilled pauses were not different in our study (matched vs. HFA), and we believe that the unfilled pauses (in our study) are primarily linked with memory retrieval. The two studies reporting significant differences both involved dialogue. However, the more naturalistic and interactive nature of dialogue necessarily means that “causes” or problems in production are more numerous. Thus, it is entirely plausible that the more naturalistic a speaking task becomes the more likely it is for different factors to “cause” problems resulting in delays. In contrast, repetitions are numerically consistent even across variable demand speaking situations, and clearly linked with speaker-internal individual differences (i.e. verbal intelligence). It is clear from the current study and several previous studies that individuals with HFA show more disfluencies in monologue tasks, and thus, their difficulties cannot simply be explained by deficits in social communication situations.

### Speaker-Versus Listener-Oriented Disfluency

Recall that Lake et al. (2011) showed a dissociation in which individuals with HFA produced fewer filled pauses and repairs, and more unfilled pauses and repetitions compared to typically-developing controls. They argued that the former are listener-oriented and the latter are speaker-oriented. The debate between speaker- and listener-oriented disfluency is important from a theoretical point of view because it focuses on what elements of speech are done for the benefit of the listener (i.e. how cooperative individuals are in dialogue). The main issue we have with the speaker versus listener conclusions of Lake et al. concerns repairs. If speakers produce repair disfluencies for the benefit of the listener, then one would expect the relationship between repairs and individual differences to be positive—higher-ability individuals should be more attuned and accommodating to listeners’ needs compared to lower-ability individuals. However, three of the five studies listed in Table B in the supplementary materials reveal the opposite pattern (i.e. ASD > TD). Moreover, the significant findings in previous work also showed negative relationships (see supplementary materials), and similar patterns were observed in the current data. We also note that the correlations between the AQ scores and repairs were mostly significant and positive. The only data point that supports the Lake et al. conclusion concerning a listener-oriented view of repairs is the study by Thurber and Tager-Flusberg, who reported means in the same direction (ASD < TD), although not significantly different (1.1. vs. 1.4).

Returning to the issue of whether repairs are listener-oriented, there is a body of work showing a lingering effect of a reparandum on comprehension (Bailey and Ferreira 2003; Ferreira and Bailey 2004; Ferreira et al. 2004; Lau and Ferreira 2005; Lowder and Ferreira 2016). That is, listeners seem to retain some representation of linguistic material that should be cancelled or eliminated by the repair. Even intuitively it is hard to imagine how a repair could be beneficial to a listener. The only explanation that makes sense is idea of (self-)correcting versus not (self-)correcting. If a speaker produces the wrong word and then does not correct their mistake, then obviously that would not be communicatively beneficial from the listener’s point of view. However, in unscripted tasks, it is difficult if not impossible to assess “non-corrected” speech errors. Because our study used a controlled speaking situation, we were able to assess what we called “recall errors” in the utterances produced, but the trends in the data were opposite of what would be expected by a self-correcting explanation (i.e. matched and unmatched controls both produced numerically more “recall errors” compared to the group with HFA).

In summary, we believe the use of clinical populations to assess theoretical questions in psycholinguistics is a good research strategy, and again, we are not in a position to make claims about filled pauses (the clearest listener-oriented disfluency) because they were not produced by speakers in our study. However, we think the idea that repairs are listener-oriented is completely unsupported given the overall trends in current data and in past research.

### Limitations and Future Directions

The obvious limitations, which affect virtually all ASD studies, are the small and heterogeneous nature of the samples. A second limitation, mentioned previously, is that we did not have an assessment of language (or reading) ability, and thus, we cannot rule out that some portion of the errors in recall performance were due to errors at encoding (i.e. reading ability). Another issue which we have discussed extensively is the controlled nature of the speaking task we used. For the types of disfluency that do not show consistent patterns across studies, these task differences make it difficult to resolve conflicting findings. In our view, it is better to work from more controlled situations and then move onto more naturalistic situations, including interactive dialogue. Perhaps production problems become more severe in cases in which the content of speech is unconstrained. If it turns out that disfluencies arise problematically within the context of unconstrained speech or in social communication, then cognitive models of language alignment become important (e.g. Pickering and Garrod 2004), and linguistic alignment has recently been investigated in autism spectrum disorders (Allen et al. 2011; Slocombe et al. 2012). For example, research is needed to establish the level at which participants with ASD fail to align. Perhaps the ideal solution is to have the same participants engage in both controlled production and naturalistic dialogue, and thus, tasks demands can be assessed within subject.^8^

## Conclusions

The aim of this study was to assess disfluency production in HFA with a view toward (1) resolving conflicting reports, (2) contributing to the literature on speaker- and listener-oriented disfluency, and (3) investigating the role of individual difference variables in the production of disfluency. We found that individuals with HFA produced more repair disfluencies and that the tendency to produce repairs is likely speaker-oriented. With respect to repetitions, we did not observe significant differences between groups, and the tendency to repeat oneself was most closely linked with verbal intelligence. Repetitions therefore, seem to be one type of disfluency that is less affected by the demands of the speaking task, but instead on a speaker-oriented individual differences variable (i.e. verbal intelligence). We also observed differences in unfilled pauses, such that the unmatched controls produced fewer unfilled pauses compared to matched controls and HFA. Unfilled pauses are somewhat subjective in nature, leading to different criteria for what actually counts as an unfilled pause between different studies. We speculated that unfilled pauses in our study were primarily due to slow memory retrieval. However, the group effect on unfilled pauses remained after covarying memory ability. It is possible that as task demands increase to be more unconstrained and interactive that individuals with HFA do in fact produce more pauses; from the present evidence, it seems highly unlikely that they produce fewer.

## Electronic supplementary material

Below is the link to the electronic supplementary material.

Supplementary material 1 (DOC 40 KB)

## Footnotes

1 We use the term “self-centric” rather than “ego-centric” throughout this paper in order to avoid any negative connotations of selfishness in interpersonal communications associated with ASD (cf. Keysar et al. 2000; Lake et al. 2011).

2 Several other studies are relevant but not applicable for various reasons. Belardi and Williams (2009) was a conference presentation that ultimately was not published. Belser and Sudhalter (2001) compared HFA with fragile X syndrome and mild mental retardation, and thus, comparisons to typically-developing individuals were not made.

3 This study contained a third group, which was referred to as “optimal outcome”. These participants had been diagnosed with an ASD, but at a later point in time, no longer showed symptoms consistent with diagnostic criteria.

4 We also coded interjections and blends. However, these affected less than ½ percent of the total utterances produced, and therefore, were excluded from all analyses.

5 The correlations for the different types of disfluency between the undergraduate coders was extremely high (i.e. presence of disfluency = 0.99, filled pause = 1.00, unfilled pause = 0.88, repetitions = 1.00, repairs = 0.94, recall errors = 0.87). Thus, we did not calculate inter-rater reliabilities. There no cases in which the third rater (i.e. the first author) disagreed with the other two coders, and so, consensus was achieved in all cases.

6 The paired comparison between the group with HFA and unmatched controls was marginally significant *t*(24) = 1.92, *p* = .07.

7 We cannot fully rule out that some errors in recall performance are due to errors at encoding (i.e. if participants misread some words, then this would obviously result in recall errors).

8 We are aware of only one study (Finlayson and Corley 2012) that compared monologue and dialogue in the same participants. However, results showed little difference in rates and types of disfluency between conditions.
