# Supplementary material for: Speaker-Versus Listener-Oriented Disfluency: A Re-examination of Arguments and Assumptions from Autism Spectrum Disorder
Source: J Autism Dev Disord. 2017 Jun 20;47(9):2885–98. doi: 10.1007/s10803-017-3215-0 (PMC5570802; doi:10.1007/s10803-017-3215-0)
Supplement: Supplementary file 1 — Supplementary material 1 (DOC 40 KB) [file 10803_2017_3215_MOESM1_ESM.doc]

Supplementary Materials

The significant correlations of the Engelhardt et al. studies are presented in Table A below. Those studies assessed various individual differences, including both performance and verbal intelligence and executive functions (i.e. inhibition and set shifting). As can be seen in the table, all of the correlations were negative, which suggests that an increased tendency to produce disfluencies is associated with lower intelligence and executive functioning. These correlations ranged from -.16 to -.32. Unfilled pauses were related to verbal intelligence. Repetitions were associated with verbal intelligence and Stroop performance. (Note that the fullscale intelligence was composed of both verbal and performance subscales of the Wechsler intelligence scale.) Repairs were associated with verbal and performance intelligence and executive functioning.

Table A

*Bivariate correlations between individual differences measures and disfluency*

Variable Unfilled Repetition Repair

1. Fullscale intelligence -.16*^a^ -.19*^a^, -.23^#c^

2. Matrix Reasoning -.27**^b^

3. Vocabulary -.22*^b^ -.26*^e^ -.22*^b^

4. Comprehension -.25*^e^

5. Information -.23*^e^

6. Similarities -.29*^e^

7. Stroop -.19*^b^ -.32**^b^, -.30**^d^

8. Perseveration -.21*^b^

*Note. ^#^p <* .08, **p* < .05, ***p* < .01. ^a^Engelhardt et al., 2010; ^b^Engelhardt et al., 2013; ^c^Engelhardt et al., 2011; ^d^Engelhardt et al., 2012; ^e^Engelhardt et al., under review.

In Table B, we have summarized the five relevant studies, including the current results. Moreover, we organized the tasks from controlled to naturalistic. The bottom part of the table lists all of the current and previous correlated variables with each type of disfluency. We struck through all results which we think do not fit or are due to a confound. Specifically, the Lake et al. (2011) results for repairs do not fit with any of the other studies in the literature, and second, given the strong relationship between verbal intelligence and repetitions, we think the Shriberg et al. and Lake et al. findings are due to not controlling for individual differences in verbal intelligence.

Table B

*Summary of HFA disfluency results*

Study Unfilled Repetitions Repairs Task

1. Current ASD>unmatTD NS ASD>TD Mono. (sentence repetition)

2. Thurber Mixed NS NS Mono. (story re-telling)

3. Suh -- ASD>TD ASD>TD Mono. (story telling)

4. Shriberg ASD>TD ~~ASD>TD~~ ASD>TD Dia. (ADOS interview)

5. Lake ASD>TD ~~ASD>TD~~ ~~ASD<TD~~ Dia. (question answering)

Correlated variables Memory (1) Verbal IQ (4) Age

ASD status

AQ scores (4)

Verbal IQ (1)

Memory (2)

Inhibition (2) as reported by Engelhardt et al.
